# Modelling of assembly error and tolerance optimization for motor based on polyhedral model and thermal deformation analysis

**DOI:** 10.1371/journal.pone.0341008

**Published:** 2026-02-05

**Authors:** Yongliang Zhang, Lulu Yang, Tianhao Ruan, Long Chen

**Affiliations:** School of Mechanical Engineering, University of Shanghai for Science and Technology, Shanghai, China; Federal University of ABC, BRAZIL

## Abstract

The air gap between the stator and rotor of an electric motor is a crucial parameter that influences its performance. A well-designed tolerance can effectively control the size of the air gap, which results from the accumulation of machining deviations. Using the air gap as an assembly functional requirement, a polyhedral model was developed to represent the assembly deviation transfer path. The effects of thermal loads under actual operating conditions were considered, and thermal deformation was incorporated into the deviation transfer path analysis. By applying Minkowski summation and intersection operations, the cumulative errors of the assembly polyhedron were determined, and the impact of thermal deformation on the assembly errors in the motor was analyzed. A multi-objective tolerance optimization model was developed, with processing and quality loss costs as the objective functions. Considering thermal deformation, a multi-objective particle swarm optimization algorithm was employed to optimize the tolerances of the motor’s critical components, providing a novel theoretical method and technical reference for assembly error analysis and tolerance optimization in electric motors.

## 1 Introduction

Permanent magnet synchronous motors (PMSMs) are widely used as effective and reliable motors with outstanding control characteristics in electric vehicles [1], industrial automation [2], and aviation applications [3]. Geometric tolerances in the process of manufacturing motors significantly affects their performance. Bramerdorfer [4] demonstrated the importance of design tolerances in influencing motor performance by analysing the tolerance sensitivity of built-in permanent magnet synchronous motors with spoke rotor structures. Han et al. [5] conducted robust optimization of surface-mounted permanent magnet synchronous motors (SPMSMs) using the Taguchi method, which confirms that the manufacturing tolerances of permanent magnets significantly affect motor performance, and emphasizes the importance of suppressing the cogging torque variations caused by such tolerances through optimized design to ensure system stability. Galfarsoro et al. [6] further confirmed that the impacts of motor manufacturing tolerances and eccentricity issues cannot be ignored by combining analytical calculations, finite element simulations, and experimental verification.

**Fig 1:**
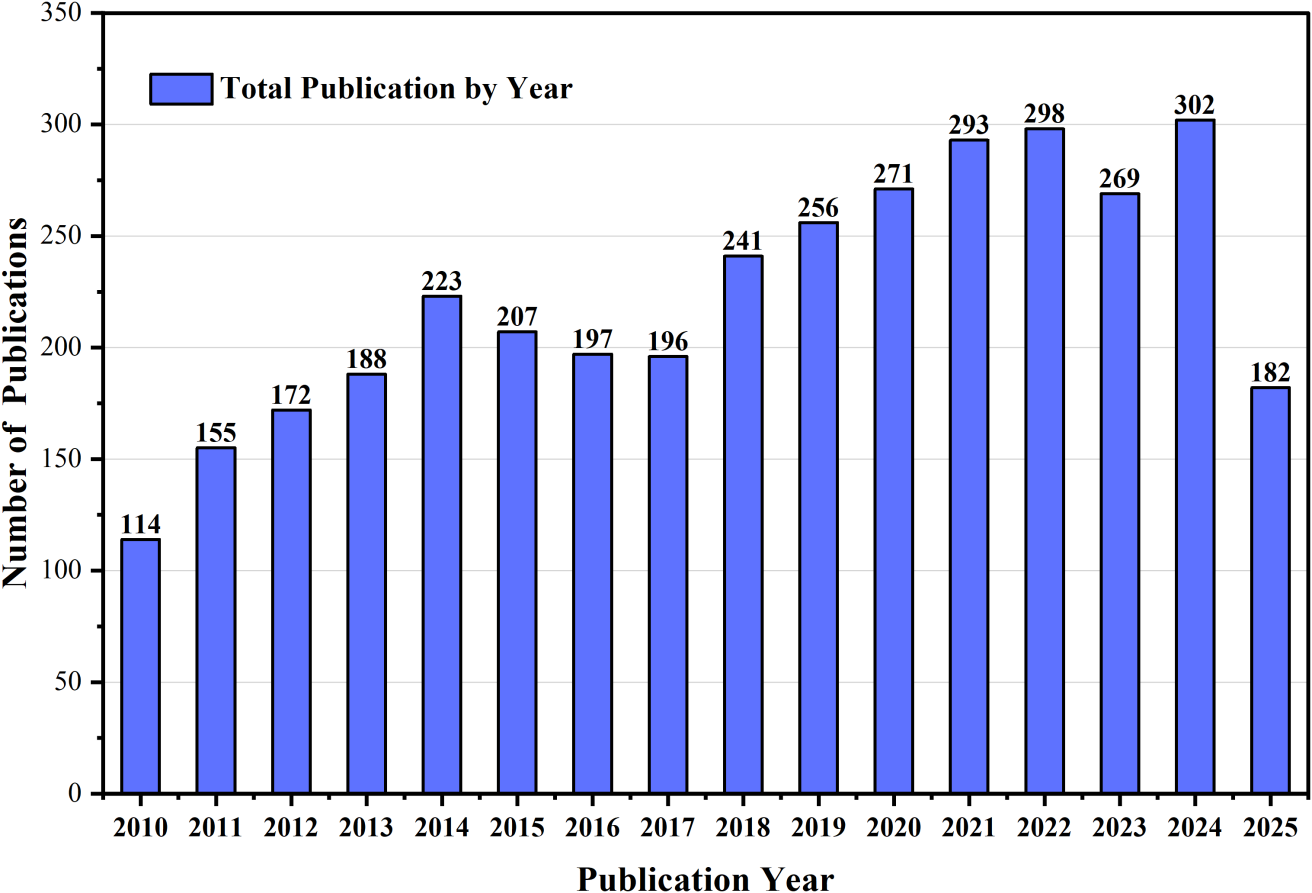
The total number of papers on tolerance design and optimization in the previous fifteen years (Web of Science, accessed on 20^th^ October 2025).

Tolerance modelling serves as the foundation for tolerance analysis and optimisation, with its core objective being to accurately characterisation of the impact of geometric deviations on the functional performance of a given product. Extensive research on tolerance analysis has been conducted both domestically and internationally. Fig 1 shows a graphical representation of publications on tolerance analysis over the past fifteen years.

Traditional tolerance design typically relies on one-dimensional or two-dimensional chain calculation methods [7], which are often inadequate for effectively representing the geometric tolerance characteristics of features and their mutual coupling relationships. To address this issue, several three-dimensional tolerance models, such as matrix [8], Jacobi [9], and Jacobi spin [10] models, have been proposed. However, these methods involve some limitations in terms of calculating the cumulative error in parallel assembly structures, and struggle to express the range of variation in the tolerance of the constituent parts of each component clearly and intuitively. In contrast, the 3D tolerance analysis method based on polyhedral modelling proposed by Teissandier [11] is applicable to all types of assemblies and can effectively analyse both series and parallel assemblies. The modelling process of this method is simpler, and the errors are intuitively visible, which provides a clear advantage for the calculations of error transfer. Liu et al. [12] proposed a polyhedral tolerance analysis model that accounts for shape defects, to further enhance the ability of the model to deal with shape defects and provide a closer approximation of real assembly conditions for tolerance design and evaluation results. Zhang et al. [13,14] and Zhi et al. [15] integrated shape deformation, manufacturing errors, and the skin model concept to significantly improve the adaptability of polyhedral models to complex assembly scenarios. García et al. [16] addressed the assemblity and functionality caused by geometric and dimensional defects of mechanical parts, and proposed a new tolerance allocation scheme for mechanical design based on a prismatic polyhedron method to address the challenges with mutual influence among tolerances and the balance between performance and cost.

In addition to geometric tolerances, the tolerance accuracy of the model depends on several factors in a real working environment. In addition to the deformation caused by force [17], the thermal load in the motor system can lead to design tolerances for parts that fail to meet the desired outcomes, which can make it more difficult to satisfy the functional requirements of a product. Using a polyhedral model, Pierre et al. [18] examined the impact of clearance and deviation on the size of assembly errors at elevated temperatures. Ge et al. [19] proposed an interactive CAD model for mechanical assembly tolerance analysis that considered manufacturing defects and the thermomechanical deformation of parts. Building on the concept of heat-solid coupling, Li et al. [20] proposed an analytical modelling technique for thermal deformation in electric spindles to generate precise offset values for thermal error correction, to improve the machining precision of the product. Although these studies provide a foundation for tolerance analysis that incorporate thermal deformation, the systematic integration of thermal effects into polyhedral modeling frameworks to analyse and optimise errors in the assembly of electric motors remains an open research challenge.

Tolerance optimisation aims to balance manufacturing costs and product performance, with its core focus being the determination of optimal tolerance allocation schemes under multi-objective constraints. Traditional methods that often rely on empirical formulas or trial-and-error approaches struggle to satisfy the design requirements for complex products. In recent years, intelligent optimisation algorithms have gradually emerged as mainstream approaches. Wang [21] comprehensively considered the relationships between productivity, production cost, machining accuracy, and scrap rate, and optimised the tolerance assignment model using the gradient descent method to minimise costs and scrap rates. Hallmann et al. [22] provided an extensive overview of tolerance-cost optimisation, identifying current challenges and future research directions. Yang et al. [23] proposed a CGA-based tolerance-cost optimization method for a multi-module spacecraft assembly, which improved accuracy and optimisation efficiency by combining comprehensive assembly precision analysis, verifying that it is superior to traditional methods and can accurately predict assembly precision. He et al. [24], Fan et al. [25], and Guo et al. [26] incorporated factors such as error sensitivity, gravity deformation, and pose error into multi-objective optimization and applied genetic algorithms, particle swarm optimization algorithms, etc., to improve the assembly precision and cost-effectiveness of precision machinery and spacecraft. One key shortcoming of the current state of the art is that conventional motor tolerance designs do not sufficiently incorporate the effects of operational temperature rise, and optimization models have yet to integrate induced assembly errors, which renders the resulting schemes poorly suited for practical operating conditions.

In summary, this study focuses on a specific company’s permanent magnet synchronous motor (PMSM) inadequate tolerance design caused by temperature rise during motor operation. Leveraging polyhedral theory, it takes the assembled motor’s air gap as a closed loop for tolerance analysis, considers thermal load under real operating conditions, simulates thermal deformation, and incorporates it into a dimensional chain analysis model to investigate thermally induced assembly errors. Furthermore, a multi-objective tolerance optimisation model is established with processing cost and quality loss cost as objectives, and economic processing capability and functional requirements of the assembly dimensional chain as the constraints to provide new theoretical and technical references for motor assembly error analysis and tolerance optimization. Finally, key components of the motor are optimised using a multi-objective particle swarm optimization (MOPSO) algorithm.

## 2 Three-dimensional tolerance modelling theory based on polyhedral models

**Fig 2:**
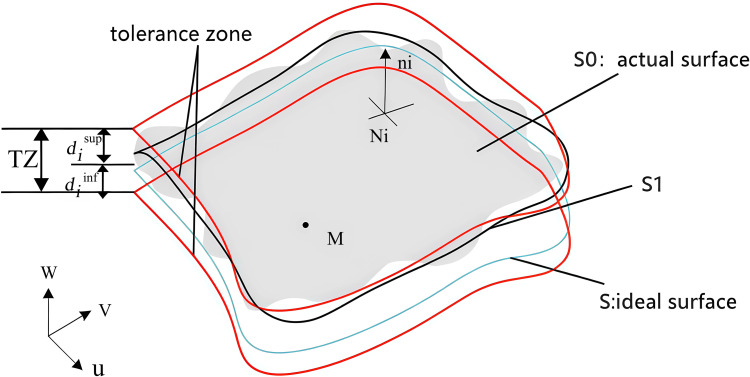
Error surface and tolerance area.

Nonlinear feature boundaries have been discretised using polyhedral models to produce linear constraints, which then create feasible domains that depict the range of feature variation. Geometric and contact polyhedra are two types of polyhedral models. Geometric polyhedra represent geometric characteristics subject to tolerance requirements, whereas contact polyhedra represent contact restrictions between mating surfaces. Using the geometric features in Fig 2 as an example, we developed a geometric polyhedral model for the geometric features of each motor component in this study, with an emphasis on describing the geometric polyhedra.

Let S be an ideal surface, and let the actual surface $S_{\text{0}}$ vary between the lower limit deviation $d_{i}^{inf}$ and the upper limit deviation $d_{i}^{sup}$. The tolerance domain for a given tolerance value is TZ[27].

An arbitrary point on the actual surface $S_{\text{0}}$ can be represented by equation (1), where $S_{\text{1}}$ is any surface in the tolerance domain,$\varepsilon_{Ni}$ is the translational displacement of $S_{\text{1}}$ with respect to ${\;S}_{\text{0}}$ at $N_{i}$; and $n_{i}$ is the normal vector at $N_{i}$.


$$S_{\text{1}}\subseteq TZ\Leftrightarrow N_{i}\in S_{\text{0}}:d_{i}^{inf}\leq \varepsilon_{Ni}\cdot n_{i}\leq d_{i}^{sup}$$ (1)

According to the theory of small-displacement spins, Eq. (1) can be expressed using equation (2) at any point M in the Euclidean space


$$d_{i}^{inf}\leq (\varepsilon_{M}+N_{M}\times \gamma )\cdot n_{i}\leq d_{i}^{sup}$$ (2)

where:$\varepsilon_{M}=(u,v,w)$ is the advective component of any point at M; r=(α,β,γ) is the rotation vector of $S_{\text{1}}$ at $N_{i}$ with respect to $S_{\text{0}}$.

The primary operations for the polyhedra are the Minkowski sum and intersection operations, and the corresponding polyhedral operations can be selected according to the assembled structure. For structures assembled in series, the Minkowski sum is used to calculate the cumulative error. For structures assembled in parallel, an intersection operation is required.

**Fig 3:**
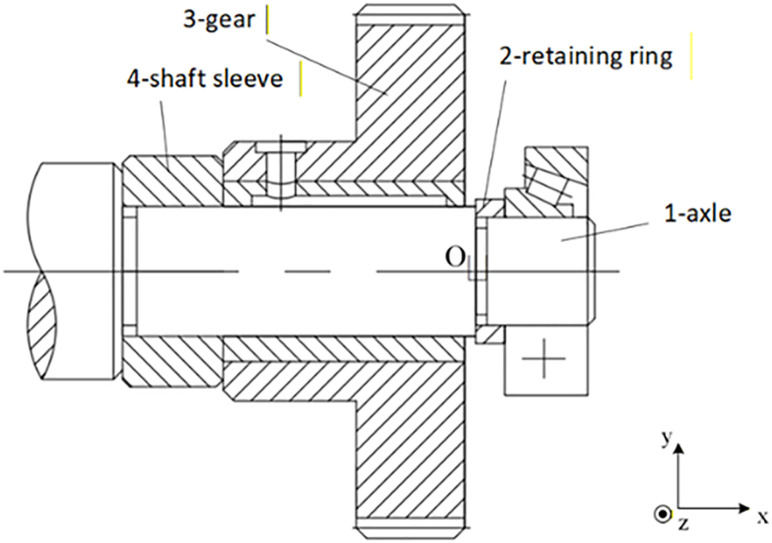
Gear mechanism.

**Fig 4:**
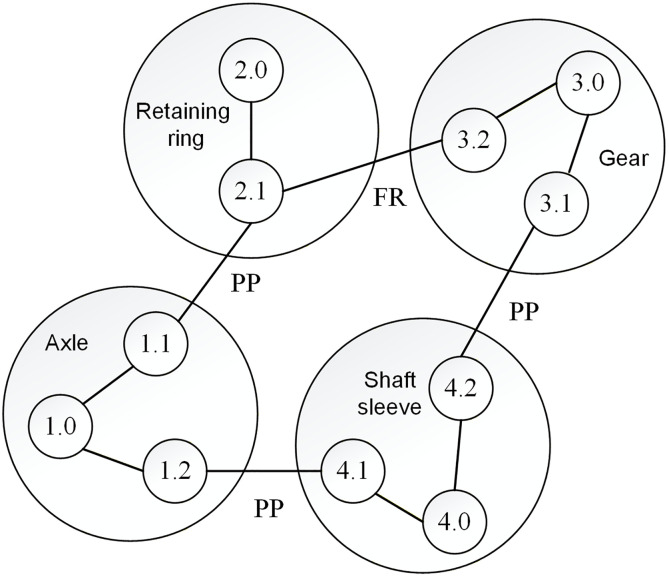
Assembly relationship diagram.

Based on these theories, the solution for the axial clearance variation in the gear assembly shown in Fig 3 can be represented by the assembly diagram in Fig 4. Component surfaces are marked as i.j, where i = 1–4 correspond to the four components, j denotes the j-th surface, and j = 0 represents the component itself. This method enables the integration of geometric and three-dimensional information into the relationship diagram. By performing Minkowski sum and intersection operations according to the series and parallel relationships of each constituent loop, more realistic results of assembly clearance variation can be obtained.

Traditional 2D tolerance analysis can’t fully capture multi-component deviation coupling. However, the polyhedron method directly models geometric deviation propagation. It quantifies how individual component variations affect assembly functions. It also improves prediction accuracy, avoids traditional empirical reliance, and provides a more intuitive, rigorous basis for tolerance allocation.

## 3 Permanent magnet synchronous motor thermal deformation analysis

The temperature of the motor increase during operation, which subjects the parts to thermal deformation and reduces the accuracy of the assembly. Therefore, the effects of thermal deformation should be considered in tolerance design. In this section, we describe how the finite element method was applied to experimentally investigate the steady-state temperature field of the motor and determine its thermal deformation under steady-state operating conditions.

### 3.1 Permanent magnet synchronous motor temperature rise experiment

**Fig 5:**
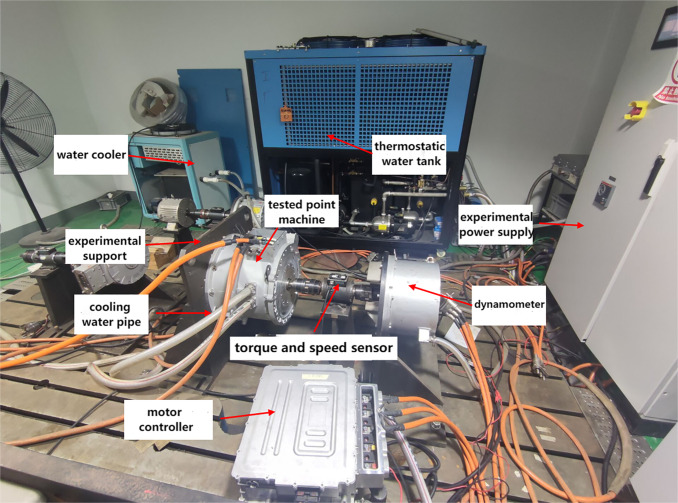
Temperature rise test platform of motor system.

The rated power of the permanent magnet synchronous motor used in this study was 40 kW, and the rated speed was 2000 rpmr. To obtain the temperature load for the thermal deformation of the motor, an experimental setup was established to measure the increase in temperature, as shown in Fig 5. A thermal imager modelled as Fluke Ti100 was used to measure the temperature of the motor’s outer surface case and end cap, and an RTD sensor modelled as NTC100K was buried in the winding in advance to measure the temperature of the motor’s internal winding. The initial temperature was held at a constant 25 °C using a chiller and a constant-temperature water tank. And after the motor operation was stabilised, the temperature measurement results of the stator winding, casing and end cover were obtained as shown in Table 1.

**Table 1:** Temperature of stator winding, casing and cap.

Type	Stator winding temperature°C	Casing temperature°C	Cap temperature°C
Actual temperature°C	76.599	31.4	33.0

### 3.2 Thermal deformation analysis of motor system

**Fig 6:**
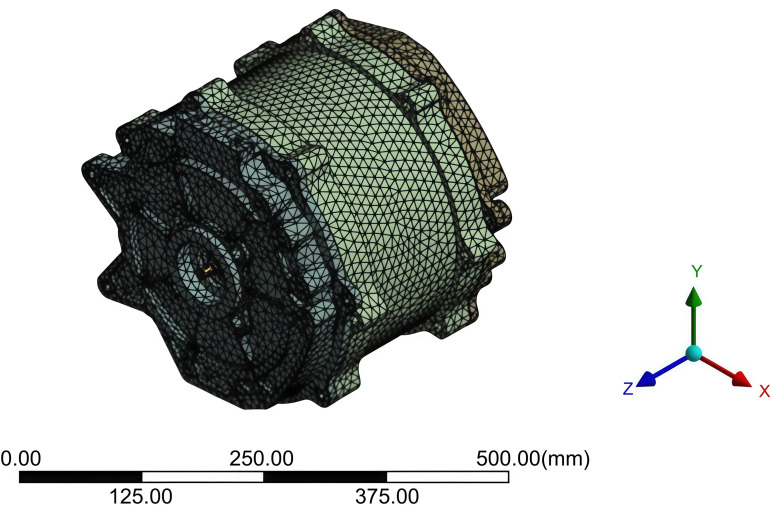
Grid division results of motor.

Tetrahedral elements were adopted for meshing. The mesh size was set to 5 mm for the left and right bearings, rotor, stator, windings, and permanent magnets, while the mesh size for other components was 20 mm. After applying the mesh, the total number of elements was 708469 and the number of nodes was 1235376, the results are shown in Fig 6.

We then sought to determine boundary conditions to solve the temperature field and thermal deformation of the motor system. We performed thermal analysis with a focus on specified temperatures and thermal convection, with key parameters calculated using empirical formulas. The heat generation rate was derived from simulated losses and the volumes of components such as the stator core and stator windings, and relevant data are presented in Table 2.

**Table 2:** Volume and heat generation rate of each motor component.

Component name	Stator core	Stator windings	Rotor	Permanent magnets	Front bearing	Rear bearing
Volume (10 ⁻ ³ m³)	2.3205	0.8169	2.1716	0.3225	0.6786	0.3699
Heat Generation Rate (10³ W/m³)	304.6663	746.4668	19.1005	270.0824	200.4126	181.1300

Regarding convective heat transfer coefficients, forced convection in water pipes was computed based on flow characteristics. Those for the stator-rotor air gap, rotor end, and rotating shaft surface were correlated with rotor speed, and a fixed combined heat transfer coefficient of 9.7 W/(m²·°C) was adopted for the housing surface and air. Both the stator windings and insulation materials were treated as homogeneous heat conductors, and the equivalent thermal conductivity of the insulation material was calculated using an empirical formula [28]. The convective heat transfer coefficients of various motor components are provided in Table 3.

**Table 3:** Calculation results of each convective heat transfer coefficient.

Parameter name	Value
Convective heat transfer coefficient between stator and rotor air gap (W/(m²·°C))	97.94
Convective heat transfer coefficient between motor housing surface and ambient air (W/(m²·°C))	9.7
Convective heat transfer coefficient between motor housing and cooling water (W/(m²·°C))	2382
Convective heat transfer coefficient between rotating shaft surface and air (W/(m²·°C))	20.17
Convective heat transfer coefficient between rotor end and air (W/(m²·°C))	94.35

**Fig 7:**
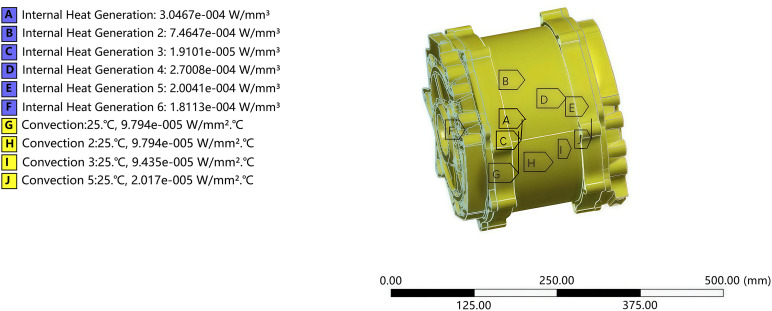
Boundary condition setting and loading result diagram of the test motor.

Based on the results of the calculation of the boundary conditions in Table 2 and 3, the heat generation rate and convective heat transfer coefficients were added as illustrated in Fig 7.

**Fig 8:**
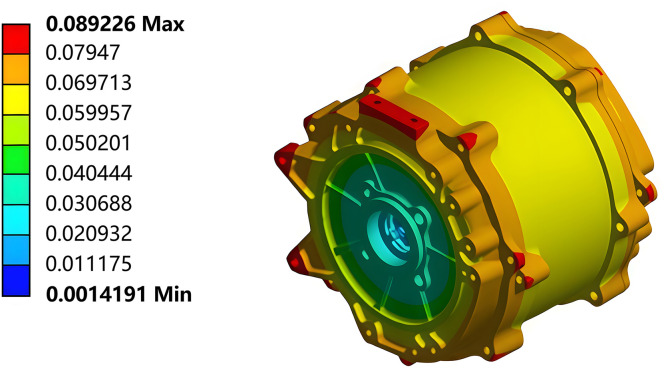
Total thermal deformation cloud diagram of the motor under steady-state operating conditions.

The steady-state temperature results of the motor were loaded into the structural analysis as thermal loads. Fixed constraints were applied to the motor casing, and the overall thermal deformation of the motor was obtained from the simulation, as shown in Fig 8.

The thermal deformation simulation results of the other key components of the motor system were obtained by applying the results of the post-processing function of the Workbench software tool. Their thermal deformation values along the X-, Y-, and Z-axes are listed in Table 4.

**Table 4:** The deformation of each part of the motor.

Parts	X-Direction (mm)	Y-Direction(mm)	Z-Direction(mm)	Placement
Rotor	0.01273	0.01103	0.00275	Rotor outer diameter surface
	0.00167	0.00261	0.00922	Rotor inner diameter surface
Stator	0.01615	0.01461	0.01704	Stator outer diameter surface
	0.02462	0.02287	0.01285	Stator inner diameter surface
Casing	0.01755	0.01783	0.00459	Inside surface of enclosure
	0.04544	0.04048	0.04102	Casing end face
	0.01571	0.01439	0.03734	Casing stop
Cap	0.04463	0.04272	0.04020	End face
	0.01418	0.01454	0.03954	End cap stop
	0.01012	0.01120	0.02316	Bearing room division
Bear	0.00892	0.00976	0.00221	Bearing outer ring
	0.00436	0.00372	0.02018	Bearing inner ring
Rotation shaft	0.00238	0.00254	0.01642	Mating with bearing
	0.00331	0.00279	0.00312	Mating with rotor

## 4 Three-dimensional assembly error analysis of motor based on polyhedral modelling

The geometrical features of the major motor components were modelled using a polyhedral model subject to tolerance constraints. We analysed the motor assembly errors under real-world operating conditions and resolved the radial variations in the motor air gap after assembly using Minkowski summation and intersection operations between the polyhedra.

### 4.1 Dimensional chain analysis for motor assembly

**Fig 9:**
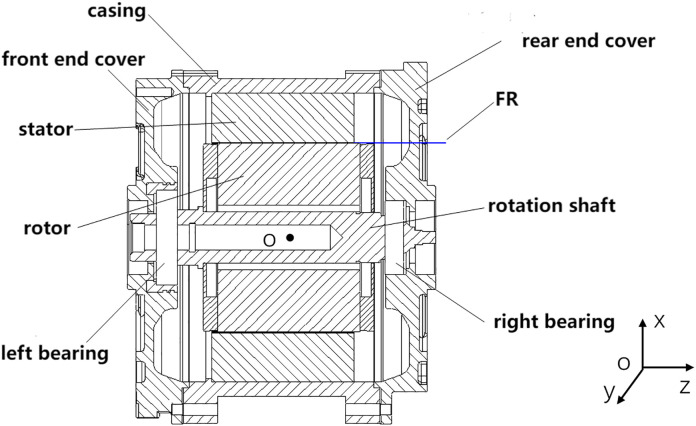
Motor assembly drawing.

In this study, the allowable variation of the air gap of the motor was set as ±0.2 mm, and the nominal size of the motor air gap was 0.75 mm. Fig 9 shows the assembly diagram of the motor, in which FR is the functional requirement of the motor assembly, that is, the air gap between the stator and rotor.

As indicated in the diagram, the parts marked 1.0, 2.0, 3.0, 4.0, 5.0, 6.0, 7.0, and 8.0 represent the stator, casing, front end cover, rear end cover, left bearing, right bearing, rotor shaft, and rotor, respectively.

**Fig 10:**
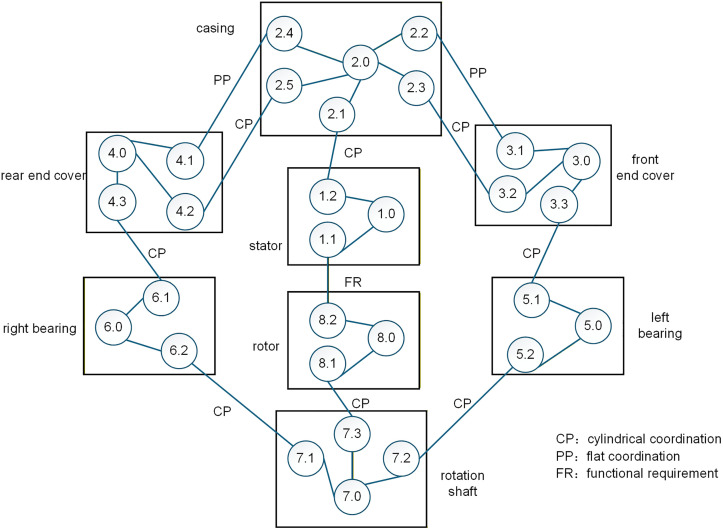
Assembly diagram. 1.1 Inner surface of stator 1.2 Outer surface of stator 2.1 Inner surface of casing 2.2 Left end surface of casing 2.3 Left stop cylindrical surface of casing 2.4 Right end surface of casing 2.5 Right stop cylindrical surface of casing 3.1 End surface of front end cover 3.2 Stop cylindrical surface of front end cover 3.3 Surface of front end cover bearing compartment 4.1 End surface of rear end cover 4.2 Rear stop cylindrical surface of rear end cover 4.3 Surface of rear end cover bearing compartment 5.1 Outer surface of left bearing 5.2 Inner surface of left bearing 6.1 Outer surface of right bearing 6.2 Inner surface of right bearing 7.1 Mating surface of rotor shaft and right bearing 7.2 Mating surface of rotor shaft and left bearing 7.3 Mating surface of rotor shaft and rotor 8.1 Inner surface of rotor 8.2 Outer surface of rotor.

**Fig 11:**
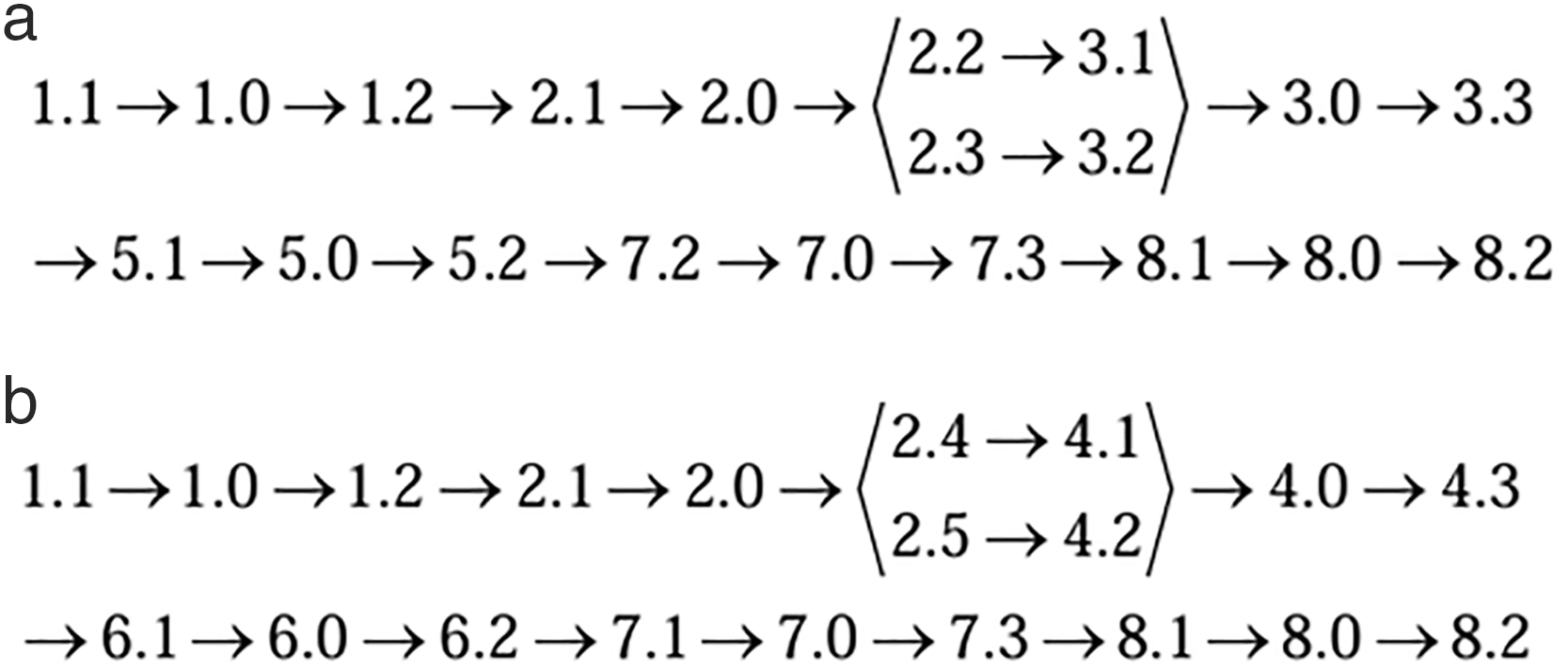
Error transfer path of air gap between features 8.2 and 1.1 in the motor assembly. (a) path Ⅰ (b) path Ⅱ.

Combined with the structure between the components in the assembly shown in Fig 10, an assembly relationship diagram of the motor assembly was established, as shown in Fig 11. From Fig 11, the air gap between features 8.2 and 1.1 in the motor assembly was obtained by the combined action of two parallel error transfer paths, denoted as paths I and II, respectively, as shown in Fig 11 (a) and (b).

Among them, 2.2 → 3.1 and 2.3 → 3.2 of path I are connected in parallel, and 2.4 → 4.1 and 2.5 → 4.2 of path II are also connected in parallel, and the overall passing from 2.0 to 7.0 is also in parallel. The other parts are in series.

If we denote by $P_{i.\frac{j}{u}.v}$ the polyhedron corresponding to each geometric feature on the transfer path, the result of the operation of polyhedral model $P_{\text{7}.\text{0}/\text{2}.\text{0}}$ on the path from 2.0 to 7.0 can be expressed as shown in equation (3).


$$P_{\text{7}.\text{0}/\text{2}.\text{0}}=(P_{\text{3}.\text{0}/\text{2}.\text{0}}⨁P_{\text{5}.\text{0}/\text{3}.\text{0}}⨁P_{\text{7}.\text{0}/\text{5}.\text{0}})⋂{(P}_{\text{4}.\text{0}/\text{2}.\text{0}}⨁P_{\text{6}.\text{0}/\text{4}.\text{0}}⨁P_{\text{7}.\text{0}/\text{6}.\text{0}})$$ (3)

among them,


$$\left\{ \begin{array}{c} P_{\text{5}.\text{0}/\text{3}.\text{0}}=P_{\text{3}.\text{3}/\text{3}.\text{0}}⨁P_{\text{5}.\text{1}/\text{3}.\text{3}}⨁P_{\text{5}.\text{0}/\text{5}.\text{1}}\\ P_{\text{7}.\text{0}/\text{5}.\text{0}}=P_{\text{5}.\text{2}/\text{5}.\text{0}}⨁P_{\text{7}.\text{2}/\text{5}.\text{2}}⨁P_{\text{7}.\text{0}/\text{7}.\text{2}}\\ P_{\text{6}.\text{0}/\text{4}.\text{0}}=P_{\text{4}.\text{3}/\text{4}.\text{0}}⨁P_{\text{6}.\text{1}/\text{4}.\text{3}}⨁P_{\text{6}.\text{0}/\text{6}.\text{1}}\\ P_{\text{7}.\text{0}/\text{6}.\text{0}}=P_{\text{6}.\text{2}/\text{6}.\text{0}}⨁P_{\text{7}.\text{1}/\text{6}.\text{2}}⨁P_{\text{7}.\text{0}/\text{7}.\text{1}} \end{array}\right.\;$$ (4)

⨁ indicates a series connection and  ⋂ indicates a parallel connection.

The result between 3.0 and 2.0 in path I and 4.0 and 2.0 in path II are respectively expressed by two sub-paths in parallel. Finally, according to the path diagram 8, on the results of each part of the operation of the series summation operation, we can obtain the expression of the functional requirements of the polyhedron $P_{\text{8}.\text{2}/\text{1}.\text{1}}$ as shown in equation (5).


$$P_{\text{8}.\text{2}/\text{1}.\text{1}}=P_{\text{1}.\text{0}/\text{1}.\text{1}}⨁P_{\text{2}.\text{0}/\text{1}.\text{0}}⨁P_{\text{7}.\text{2}/\text{2}.\text{0}}⨁P_{\text{8}.\text{0}/\text{7}.\text{0}}⨁P_{\text{8}.\text{2}/\text{8}.\text{0}}$$ (5)

among them,


$$\left\{ \begin{array}{c} P_{\text{2}.\text{0}/\text{1}.\text{0}}=P_{\text{1}.\text{2}/\text{1}.\text{0}}⨁P_{\text{2}.\text{1}/\text{1}.\text{2}}⨁P_{\text{2}.\text{0}/\text{2}.\text{1}}\\ P_{\text{8}.\text{0}/\text{7}.\text{0}}=P_{\text{7}.\text{3}/\text{7}.\text{0}}⨁P_{\text{8}.\text{1}/\text{7}.\text{3}}⨁P_{\text{8}.\text{0}/\text{8}.\text{1}} \end{array}\right.\;$$ (6)

### 4.2 Calculation of cumulative error for functional requirements based on thermal deformation

The tolerances of the relevant feature surfaces of the motor were corrected based on the thermal deformation of each part obtained from Table 2, and the results are listed in Table 5.

**Table 5:** Tolerance information after considering thermal deformation.

Symbolic representation	t_1.1_	t_1.2_	t_2.1_	t_2.2_	t_2.3_	t_3.1_	t_3.2_	t_3.3_	t_5.1_	t_5.2_	t_7.2_	t_7.3_	t_8.1_	t_8.2_
Original tolerance(mm)	0.03	0.04	0.03	0.08	0.05	0.08	0.06	0.08	0.06	0.05	0.03	0.03	0.04	0.04
Corrected tolerance(mm)	0.0546	0.0562	0.0476	0.1210	0.0657	0.1202	0.0742	0.0901	0.0689	0.0547	0.0324	0.0333	0.0417	0.0513

Note: t_1.1_ and t_1.2_ are expressed as the stator bore runout and outer diameter cylindricity; t_2.1_, t_2.2_ and t_2.3_ are expressed as the casing inner diameter cylindricity, casing stop end face runout, and radial runout; t_3.1_, t_3.2,_ and t_3.3_ are expressed as the axial and radial runouts and cylindricity of the end cover stop; t_5.1_ and t_5.2_ are expressed as the radial and axial runouts of the bearing; t_7.2_, t_7.3_, and t_8.1_, t_8.2_ are expressed as the rotor cylindricity and radial runout, respectively.

**Fig 12:**
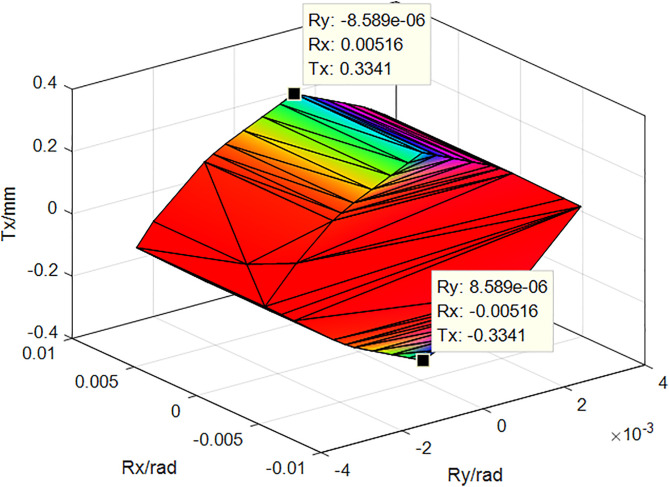
Functional requirement polyhedron ${\;𝐏}_{8.2/1.1}$ of motor assembly considering thermal deformation.

The corrected tolerance values listed in Table 3 were substituted into equation (2), to establish the corresponding constraints are established. The geometrical characteristics of polyhedra, such as $P_{\text{1}.\text{0}/\text{1}.\text{1}}$in the error transfer path shown in Fig 12, were then determined computationally. These values were subsequently substituted into equation (5) to obtain the functionally required polyhedra $P_{\text{8}.\text{2}/\text{1}.\text{1}}$, as shown in Fig 12. In the figure, Tx, Rx, and Ry are the translation error in the x-direction, rotation error in the X-direction, and rotation error in the Y-direction, respectively.

**Fig 13:**
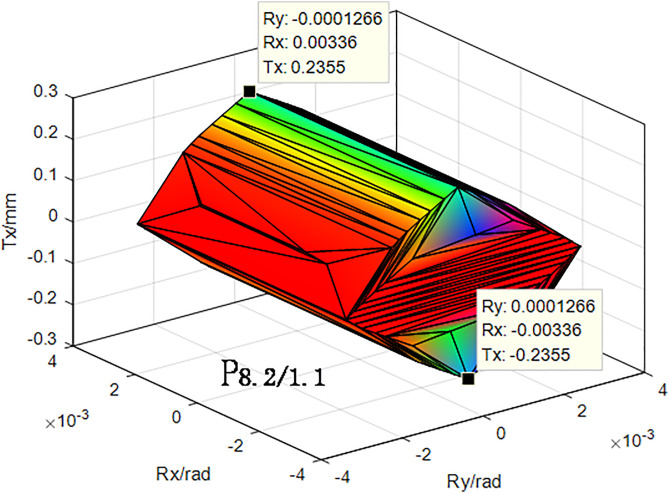
Ideal functional requirement polyhedron${\;𝐏}_{\frac{8.2}{1.1}}$ of the motor assembly without considering thermal deformation.

To evaluate the effects of thermal deformation on the tolerance design of the motor, an ideal polyhedron that did not consider thermal deformation was calculated, as shown in Fig 13.

The cumulative error of the assembly can indicate the variation of the error of the assembly in each direction. The range of variation of the translation error of the assembly along the X-axis shown in Fig 12 was ± 0.3341 mm, the range of the rotation around the X-axis was ± 0.00516 rad, and the range of the rotation around the Y-axis was ± 8.589 × 10−6 rad. It may be observed that the translation error around the X-axis is the largest, and this was the main factor causing an unreasonably large air gap between the assembled rotor and stator. The size of the air gap between the assembled rotor and stator was determined to be unreasonable. Comparing the data in Figs 12 and 13, the unilateral assembly error of the X-direction translation increased from 0.2355 to 0.3341 mm, and the total error increases by 0.1972 mm, which was 41.86% of the total error without considering thermal deformation. This indicates that the actual operating conditions significantly affected the results of the tolerance analysis.

## 5 Optimized design of motor tolerance based on particle swarm algorithm

A multi-objective tolerance optimisation model was established and solved using the particle swarm algorithm to realise the multi-objective tolerance optimisation of the motor system to ensure assembly quality and reduce manufacturing costs based on the analysis of errors in the motor assembly model considering thermal deformation.

### 5.1 Establishment of multi-objective optimization model for motor tolerance

Considering the processing and quality loss costs, the objective function was determined as shown in equation (7).


$$\left\{ \begin{array}{c} minC=\sum_{i=\text{1}}^{\text{14}}C\left(t_{i}\right)\\ minL=\text{343}.\text{75}\cdot \sum_{i=\text{1}}^{\text{14}}t_{i}^{\text{2}} \end{array}\right.\;$$ (7)

$$\begin{array}{cc} C\left(t_{i}\right)= & \;\text{0}.\text{0}\text{3}\text{7}\text{3}e^{-\text{3}.\text{0}\text{8}t_{\text{1}.\text{1}}}+\text{1}\text{5}.\text{1}\text{1}\text{3}e^{-\text{4}\text{2}.\text{2}\text{8}\text{7}\text{4}t_{\text{1}.\text{2}}}+\frac{t_{\text{1}.\text{2}}}{\text{0}.\text{8}\text{6}\text{1}\text{1}t_{\text{1}.\text{2}}+\text{0}.\text{0}\text{1}\text{5}\text{0}\text{8}}\\ & \;+\text{1}\text{2}.\text{6}\text{6}\text{9}\text{1}e^{-\text{3}\text{7}.\text{5}\text{2}\text{7}\text{9}t_{\text{2}.\text{1}}}+\text{2}.\text{4}\text{8}\text{6}e^{\frac{\text{0}.\text{0}\text{0}\text{0}\text{9}\text{7}\text{8}}{t_{\text{2}.\text{1}}}}+\text{0}.\text{0}\text{3}\text{7}\text{3}e^{-\text{3}.\text{0}\text{8}t_{\text{2}.\text{2}}}+\text{0}.\text{0}\text{3}\text{7}\text{3}e^{-\text{3}.\text{0}\text{8}t_{\text{2}.\text{3}}}+\text{0}.\text{0}\text{3}\text{7}\text{3}e^{-\text{3}.\text{0}\text{8}t_{\text{3}.\text{1}}}\\ & \;+\text{0}.\text{0}\text{3}\text{7}\text{3}e^{-\text{3}.\text{0}\text{8}t_{\text{3}.\text{2}}}+\text{1}\text{2}.\text{6}\text{6}\text{9}\text{1}e^{-\text{3}\text{7}.\text{5}\text{2}\text{7}\text{9}t_{\text{3}.\text{3}}}+\text{2}.\text{4}\text{8}\text{6}e^{\frac{\text{0}.\text{0}\text{0}\text{0}\text{9}\text{7}\text{8}}{t_{\text{3}.\text{3}}}}\\ & \;+\text{0}.\text{0}\text{3}\text{7}\text{3}e^{-\text{3}.\text{0}\text{8}t_{\text{5}.\text{1}}}+\text{2}.\text{7}\text{8}\text{4}e^{-\text{3}\text{6}.\text{6}\text{1}t_{\text{5}.\text{2}}}+\text{1}.\text{1}\text{2}\text{5}e^{\frac{\text{0}.\text{0}\text{0}\text{7}\text{5}}{t_{\text{5}.\text{2}}}}\\ & \;+\text{0}.\text{0}\text{3}\text{7}\text{3}e^{-\text{3}.\text{0}\text{8}t_{\text{7}.\text{2}}}+\text{0}.\text{0}\text{3}\text{7}\text{3}e^{-\text{3}.\text{0}\text{8}t_{\text{7}.\text{3}}}+\text{1}\text{2}.\text{6}\text{6}\text{9}\text{1}e^{-\text{3}\text{7}.\text{5}\text{2}\text{7}\text{9}t_{\text{8}.\text{1}}}\\ & \;+\text{2}.\text{4}\text{8}\text{6}e^{\frac{\text{0}.\text{0}\text{0}\text{0}\text{9}\text{7}\text{8}}{t_{\text{8}.\text{1}}}}+\text{0}.\text{0}\text{3}\text{7}\text{3}e^{-\text{3}.\text{0}\text{8}t_{\text{8}.\text{2}}} \end{array}$$ (8)

Considering the economic machining capacity and assembly function requirements, constraints were established as shown in equation (9).


$$s.t\left\{ \begin{array}{c} \sqrt{\sum_{i=\text{1}}^{\text{14}}\xi_{i}^{\text{2}}t_{i}^{\text{2}}\leq \text{0}.\text{4}}\\ t_{imin}\leq t_{i}\leq t_{imax} \end{array}\right.\;$$ (9)

For the assembly tolerance optimisation design, the number of constituent rings was set as the dimensions of the particle solution space, and the tolerance value was used as the particle position component. The upper and lower limits of the particle search space and the velocity value interval were determined based on the tolerance range. to accelerate the convergence to the global optimum, and the particle velocity was updated by decreasing the inertia weights. The parameters of the algorithm are listed in Table 6.

**Table 6:** MOPSO algorithm control parameter setting.

Population size	Iteration number	Learning factor${\;C}_{\text{1}}$	Learning factor${\;C}_{\text{2}}$	Inertia weight ω
200	100	1. 5	1. 5	0.4 ～ 0.9

### 5.2 Optimization result analysis

**Fig 14:**
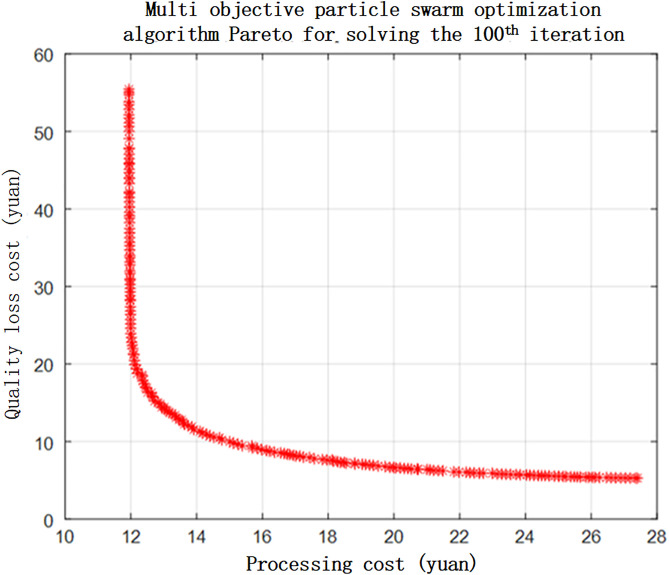
Machining cost and quality loss cost optimization Pareto frontier of machining cost and quality loss cost in motor tolerance optimization.

The multi-objective particle swarm algorithm was used to optimally solve the established multi-objective multi-constraint model of the motor. After 100 iterations, the Pareto front of the motor model was solved, as shown in Fig 14.

According to Fig 14, the machining cost should be between 12 and 16 to optimise the tolerance of each constituent ring. According to the Pareto solution set, the optimum tolerance values for each constituent ring were obtained by considering the quality loss and machining cost. The optimised tolerance values were revised according to the national standards, and the tolerance values before and after optimisation are listed in Table 7.

**Table 7:** Tolerance optimization result.

Tolerance	t_1.1_	t_1.2_	t_2.1_	t_2.2_	t_2.3_	t_3.1_	t_3.2_	t_3.3_	t_5.1_	t_5.2_	t_7.2_	t_7.3_	t_8.1_	t_8.2_
Optimized results/mm	0.02	0.05	0.03	0.05	0.03	0.06	0.03	0.06	0.03	0.08	0.02	0.02	0.06	0.03

**Fig 15:**
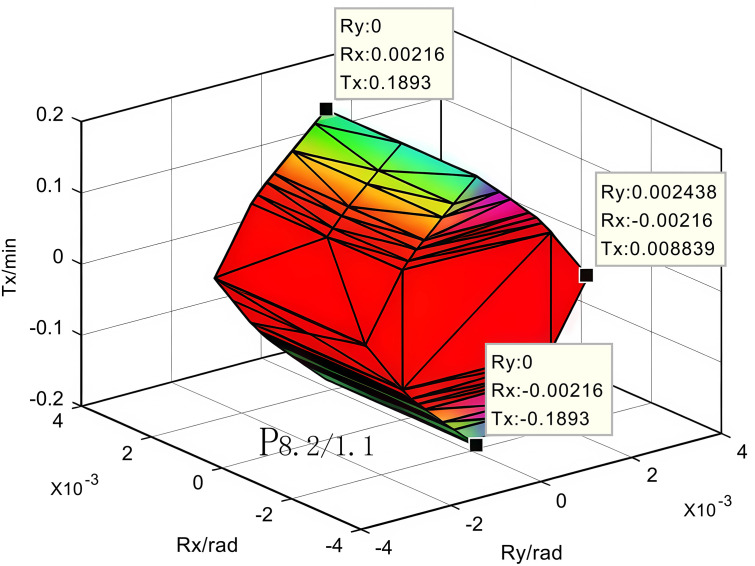
Optimized polyhedron of motor assembly error.

According to the optimised tolerance values, the upper and lower boundary parameters of the constraint equations were corrected to obtain the corresponding optimised polyhedra and establish the optimised polyhedral model of errors in the motor assembly, as shown in Fig 15.

Comparison of the results in Fig 15 and Fig 12 shows that the X-direction assembly error before and after optimisation decreased from 0.3341 mm to 0.1893 mm, a reduction of 0.1448 mm, which was a total of 43.3%, and meets the assembly requirement of ±0.2 mm. The original tolerance, heat distortion correction tolerance, and optimisation tolerance were compared as shown in Table 8. By comparing the results with those of methods such as [2] and [3], it was demonstrated that the calculation method using the polyhedral model considering thermal deformation yielded smaller errors.

**Table 8:** Assembly error value.

Name	Maximum Value/mm	Minimum Value/mm	Error/mm
Original tolerance	0.2355	−0.2355	0.4710
Heat distortion correction tolerance	0.3341	−0.3341	0.6682
Optimisation tolerance	0.1893	−0.1893	0.3786

**Fig 16:**
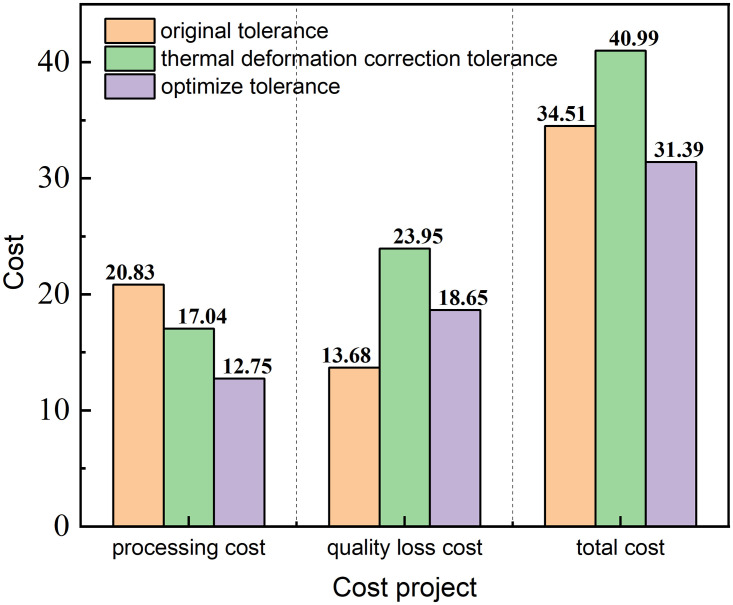
Optimization before and after cost comparison.

These values were then substituted into the objective function, and the cost results for the three cases were calculated, as shown in Fig 16.

As illustrated in Fig 16 after considering the impact of thermal deformation, the total cost of the motor increased from 34.51 CNY to 40.99, representing an increase by 18.78%. Following algorithmic optimisation, the total cost was reduced to 31.39 CNY, resulting in decreases of 4.29, 5.30, and 9.60 in processing costs, quality loss costs, and total costs (all in CNY), due to thermal deformation. These reductions correspond to 25.18%, 22.13%, and 23.42%, respectively.

These results show that after considering the thermal deformation introduced by the actual operating conditions, if the tolerance design were not optimised, the total cost would be significantly increased, whereas the optimised tolerance can ensure machining quality and consider costs realistically to obtain the best technical and economic benefits.

### 5.3 Tolerance-cost sensitivity analysis

To verify the economic robustness of the optimized tolerance scheme, a single-factor sensitivity analysis was conducted based on the cost function established in Section 5.1 and the optimization results. Among the 14 core tolerances, 13 were fixed at their optimized values, while only the target tolerance was independently perturbed within the feasible range. Calculated the relative change rate of total cost under different perturbations, expressed as $C_{total}/C_{total.o}$. $C_{total.o}$ is the total cost of the optimized scheme — the optimized total cost is 31.39 CNY. $C_{total}$ is the total assembly and production cost after independently perturbing the target tolerance. The calculate results for the 14 tolerances are listed in Table 9.

**Table 9:** Motor core tolerance-cost sensitivity analysis results.

Tolerance	t_1.1_	t_1.2_	t_2.1_	t_2.2_	t_2.3_	t_3.1_	t_3.2_	t_3.3_	t_4.1_	t_5.1_	t_6.1_	t_7.1_	t_8.1_	t_8.2_
Relative Change Rate (%)	7.25	3.68	3.42	6.89	1.91	2.95	1.87	1.78	3.34	1.65	6.12	4.89	3.57	6.58

The results of the calculation showed that among the 14 tolerances, 10 items exceed 2%, with key items including t_1.2_, t_2.2_, and t_6.1_ surpassing 5%. This demonstrates that these tolerances exert a valid impact on the results. While the remaining four are below 2%, they are indispensable components of the analysis. Sensitivity analysis indicates that the tolerance items selected for analysis in this study are reasonable and convincing.

## 6 Conclusions

In this study, considering the influence of thermal deformation on the assembly accuracy of the motor, assembly errors of a permanent magnet synchronous motor have been analysed based on the polyhedron theory. And the multi-objective particle swarm algorithm was used to optimize the geometrical tolerance of the key constituent rings of the motor. The results of this study not only clarify the technical details but also reveal the values of applying this method more broadly. Our key findings are summarized below.

(1) Motor assembly error analysis was performed using a polyhedral model, and the motor assembly error transfer path was identified. The Minkowski sum and intersection operation are used to express the connection relationship of the series-parallel connections of the assembly parts. Its core logic can be directly transferred to the assembly analysis of other rotating electrical machines such as asynchronous motors and switched reluctance motors.
(2) To determine the thermal deformation caused by the motor heat, a temperature-rise test platform was built, and the temperatures of every component of the motor were recorded while it was operating normally. The results of the temperature analysis were then incorporated into the structural analysis as thermal loads.
(3) A polyhedral model of the motor assembly tolerance under actual operating conditions was established, the stator-rotor air gap assembly error of the motor was modelled and analysed, the thermal deformation was integrated into the analysis of the assembly error under actual operating conditions, and the assembly error was analysed through simulation.
(4) A multi-objective particle swarm optimisation method was employed to address the tolerance optimisation model. The results indicate that the airgap assembly error post-optimisation decreases by 0.1448 mm, which is equivalent to a 43.3% reduction, thereby meeting the functional requirements of the assembly. Furthermore, there was a decrease in machining cost by 4.29 CNY, quality loss cost by 5.30 CNY, and an overall cost reduction of 9.60 CNY.

In summary, the heat-error coupling analysis and optimization framework established in this study for permanent magnet synchronous motors provides a method for precision assembly tolerance design that considers the influence of operating conditions. This method can be applied to improve the assembly accuracy of various types of motors. Moreover, this work also provides a replicable analytical paradigm for tolerance optimization in the entire high-end equipment manufacturing. In the future, we expect that the application scenarios of the method applied here will be further extended to the field of precision manufacturing under extreme operating conditions by incorporating dynamic thermal loads and material aging factors.
